# The influence of contrast media on calcium-based imaging of the spine in dual-layer CT

**DOI:** 10.1038/s41598-024-69743-3

**Published:** 2024-08-14

**Authors:** S. Rahn, S. Skornitzke, C. Melzig, T. Reiner, W. Stiller, C. P. Heussel, H. U. Kauczor, T. F. Weber, T. D. Do

**Affiliations:** 1 Clinic of Diagnostic and Interventional Radiology (DIR), Heidelberg University Hospital, Im Neuenheimer Feld 420, 69120 Heidelberg, Germany; 2 Clinic of Orthopedics and Trauma Surgery, Heidelberg University Hospital, Heidelberg, Germany; 3 Department of Radiology, Thoraxklinik Heidelberg, Heidelberg University Hospital, Heidelberg, Germany

**Keywords:** Computed tomography, Virtual non-calcium, Dual-layer spectral CT, Contrast media, Clinical trials, Cancer, Endocrine system and metabolic diseases

## Abstract

This study aimed to evaluate the impact of contrast media application on CT attenuation of the bone using a novel calcium-only imaging technique (VCa) from dual-layer spectral detector CT (DLCT), which enables CT-based bone mineral density measurement unimpeded by soft tissue components. For this, true non-contrast (TNC) and venous phase images (VP) of *n* = 97 patients were acquired. CT attenuation of the first lumbar vertebra (L1) was measured in TNC-VCa, VP-VCa, and in virtual non-contrast images (VNC). CT attenuation was significantly higher in VP-VCa than in TNC-VCa (*p* < 0.001), although regression analyses revealed a strong linear association between these measures (*R*^2^ = 0.84). A statistical model for the prediction of TNC-VCa CT attenuation was established (TNC-VCa[HU] = − 6.81 + 0.87 × VP-VCa[HU]-0.55 × body weight[kg]) and yielded good agreement between observed and predicted values. Furthermore, a L1 CT attenuation threshold of 293 HU in VP-VCa showed a sensitivity of 90% and a specificity of 96% for detecting osteoporosis. The application of contrast media leads to an overestimation of L1 CT attenuation in VCa. However, CT attenuation values from VP-VCa can be used within CT-based opportunistic osteoporosis screening eighter by applying a separate threshold of 293 HU or by converting measured data to TNC-VCa CT attenuation with the given regression equation.

## Introduction

Osteoporosis, characterized by low bone mass and deterioration of bone microarchitecture, leads to a notable increase in fracture risk^1,2^. Despite its substantial impact on morbidity, mortality and healthcare expenditures^3,4^ osteoporosis remains underdiagnosed and undertreated^5–7^. Early detection is crucial for effective treatment and fracture prevention, prompting research into the use of routine computed tomography (CT) for opportunistic osteoporosis screening^8–11^. Conventional single-energy CT (SECT) has broad screening potential^12,13^ in evaluating bone mineral density (BMD), but these estimates are prone to errors due to contributions from both bone and soft tissue. Thus, bone marrow changes (e.g., due to conversion of red bone marrow to bone marrow fat or tumor infiltration) could lead to a systematic over- or underestimation of BMD^9,14–17^. Dual-energy CT (DECT) offers a solution by differentiating bone from soft tissue^15,18–20^. The introduction of the Dual-layer spectral detector CT (DLCT) scanner, which uses two separate detector layers to capture different parts of the X-ray spectrum, has advanced the clinical use of energy-resolved imaging without the need for specific dual-energy protocols or increased radiation exposure^21^. Thus, with DLCT, spectral data can be obtained without pre-selecting a particular dual-energy protocol or increasing radiation exposure.

A recent study utilizing spectral data from DLCT has introduced a calcium-based postprocessing algorithm. This technique facilitates the reconstruction of virtual calcium-only images (VCa), which depict bone structures devoid of soft tissue interference^22^. In that investigation, CT attenuation values in VCa demonstrated superior concordance with DXA-derived bone mineral density (BMD) and known calcium concentrations compared to conventional CT images. This suggests that VCa could enhance the accuracy of CT-based osteoporosis screening.

Considering that the previous study encompassed only non-contrast examinations and recognizing that iodinated contrast media (CM) significantly influence CT attenuation of bone tissue, the applicability of the findings is confined to non-enhanced DLCT data^22–25^. Consequently, this study aimed to assess whether BMD can be accurately measured in VCa reconstructed from contrast-enhanced CT data. Given that the majority of clinical CT scans involve CM administration, evaluating the feasibility of calcium-only imaging for an opportunistic osteoporosis screening necessitates this investigation. Additionally, a secondary objective was to assess the utility of virtual non-contrast reconstructions derived from post-contrast imaging.

## Material and methods

### Ethics approval and consent

This retrospective exploratory single-center study was approved by the loval review board ethics committee of the medical faculty of Heidelberg University (S-348/2019). The need for written informed consent was waived. The study was performed according to the ethical guidelines of the 1964 Declaration of Helsinki and its later amendments.

### Study design and clinical data selection

The primary objectives were:

1. to compare mean CT attenuation values of the first lumbar vertebra (L1) measured in VCa of true non-contrast acquisitions (TNC-VCa) with mean CT attenuation values of L1 measured in VCa of post-contrast venous phase acquisitions (VP-VCa)
2. to establish a statistical model enabling the prediction of TNC-VCa L1 CT attenuation values from VP-VCa L1 CT attenuation values.

Secondary objectives were:

1. to compare mean CT attenuation values of L1 between the following reconstructions:
  - conventional images reconstructed from true non-contrast acquisitions (TNC) vs. virtual non-contrast images reconstructed from post-contrast venous phase acquisitions (VNC),
  - TNC vs. post-contrast venous phase acquisitions (VP),
  - TNC vs. TNC-VCa
  - VP vs. VP-VCa
2. to assess the capability of VP-VCa L1 CT attenuation values in detecting osteoporosis.

Osteoporosis was defined as a L1 CT attenuation value ≤ 110 HU in TNC images as suggested by Pickhardt et al.^11^.

**Figure 1:**
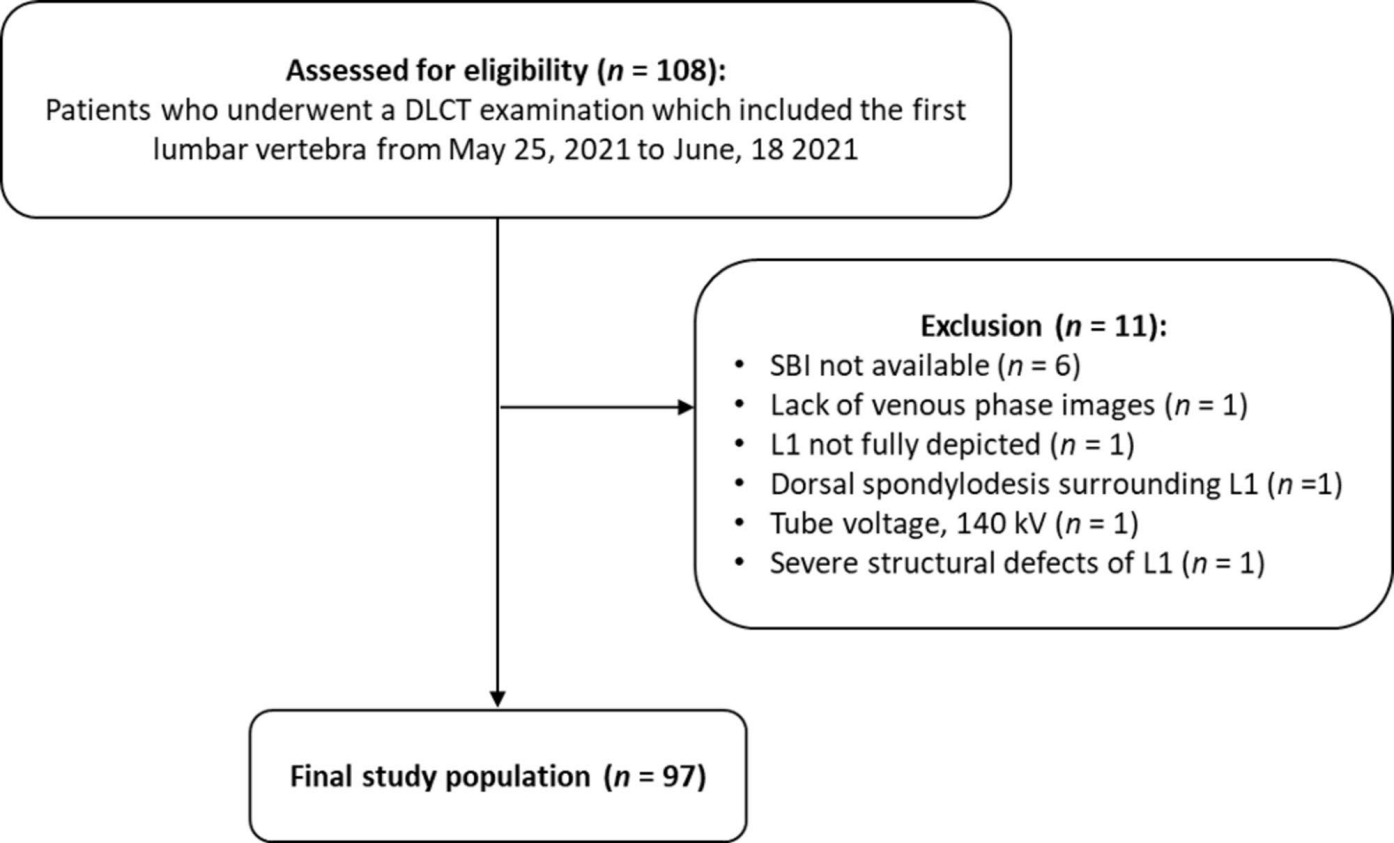
Flow chart of study enrollment in line with the STROBE guidelines^38^. *DLCT* dual-layer spectral detector CT, *L1* first lumbar vertebra, *SBI* spectral base images.

For this purpose, all patients of Heidelberg University Hospital who underwent a DLCT examination including the thoracolumbar junction from May 25, 2021 to June, 18 2021 were considered for study inclusion. In total, *n* = 108 patients were identified. Inclusion criteria were: (1) availability of a DLCT examination including a true non-contrast and a post-contrast venous phase acquisition of the thoracolumbar junction, (2) availability of spectral base images (SBI) for both acquisitions to enable the reconstruction of virtual non-contrast images from venous phase acquisitions and calcium-suppressed images from both acquisitions, (3) age at least 18 years. Criteria for study exclusion were: (1) severe structural defects of L1 such as fractures (Genant grade 2 and 3) and malignant lesions, (2) presence of dorsal spondylodesis, cement augmentation, or (3) severe imaging artifacts. The final study population consisted of *n* = 97 patients. The process of study enrollment is depicted in Fig. 1. Clinical information on patient age, sex and body weight were extracted from the hospital information system (I.S.-H.*med., SAP).

### DLCT acquisition and post-processing

A dual-layer spectral CT (Spectral CT7500, Philips Healthcare) with helical technique was used for the imaging of all patients. All acquisitions were performed with the same parameters: Tube voltage 120 kV_p_, dose right index 18 (automated, attenuation-based dose modulation), reference tube current–time product 87 mAs, volumetric computed tomography dose index (CTDI_vol_) 7.5 mGy (32 cm body-phantom), pitch 1.473 and collimation 64 × 0.625 mm. For contrast enhancement, all patients received an individually tailored body weight-adapted CM bolus using a dedicated contrast injection software (Certegra™ P3T, Bayer). The calculation of the amount of contrast media was as followed:

$$ {\text{Contrast}}\,{\text{media}}\,{\text{volume }}\left[ {{\text{ml}}} \right]\, = \,{\text{body}}\,{\text{weight}}\, \times \,0.4/{\text{iodine}}\,{\text{concentration}}\, \times \,1000. $$

### Image reconstruction and analysis

Using the manufacturer’s image post-processing suite (IntelliSpace Portal Version 12, Philips), overlapping sagittal reconstructions of the lumbar spine were computed with 2 mm slice thickness and 1 mm increment. The following reconstructions were created from SBI of true non-contrast acquisitions: (1) conventional images (TNC), (2) virtual non-Calcium images, suppression index 25 (VNCa25), and (3) virtual non-Calcium images, suppression index 100 (VNCa100). Additionally, from SBI of post-contrast venous phase acquisitions, the following reconstructions were created: (1) conventional images (VP), (2) virtual non-contrast images (VNC), (3) virtual non-Calcium images, suppression index 25 (VNCa25), and (4) virtual non-Calcium images, suppression index 100 (VNCa100). For TNC and VP reconstructions, a sharp kernel YA and for VNCa reconstructions kernel B was used. The higher the VNCa index in the reconstruction the less calcium was subtracted and more bony structures were visible. VCa CT numbers were calculated as difference between VNCa100 and VNCa25 CT attenuation values and therefore are considered to be a measure of bone mass which is supposed to be unaffected by soft tissue components.

Using the same software as for image reconstruction, oval ROIs as large as possible were placed in the center of L1, avoiding the inclusion of blood vessels or cortical bone. The focus was set on L1 as (1) it is included on many routine CT examinations (like abdomen and thorax), (2) can be easily identified, and (3) research results suggest that BMD measurements of L1 are as accurate or more accurate than those of other thoracal or lumbar vertebrae or even than combinations of multiple vertebrae^11,26,27^. For all reconstructions of the true non-contrast and the contrast-enhanced acquisition, respectively, the positions of the ROIs were identical to ensure comparability of measurements. The ROI measurements were performed by two independent readers to increase accuracy and to quantify interreader agreement. For the main statistical analyses, mean CT attenuation values of both reads were calculated.

### Statistical analysis

The statistical analyses were performed in the statistical environment R (version 4.1.1)^28^. In case of multiple comparisons, the Bonferroni-Holm method was used for p-value adjustment^29^. The level of significance was set at *p* < 0.05. The intra-class correlation coefficient (ICC) with corresponding 95% confidence intervals was calculated as a measure of interrater reliability basing on single-rater [k = 2], absolute agreement, 2-way random effects models^30^. Wilcoxon Signed-Rank tests were performed to compare CT attenuation values of L1 in TNC-VCa, VP-VCa, TNC, VP and VNC images; corresponding effect sizes are reported as *r* = *Z*/$\sqrt{N}$^31^. In order to establish a statistical model for the prediction of TNC-VCa CT numbers from VP-VCa CT numbers, linear regression analysis was conducted with TNC-VCa CT numbers as dependent variable. In a first step, a univariate regression model was fitted using VP-VCa CT attenuation values as predictor to determine the linear association between TNC-VCa and VP-VCa CT attenuation. In a second step, multiple regression analysis with backwards elimination of predictors was performed including age, sex and body weight as further potential predictors of TNC-VCa CT numbers. Assessing the regression models according to standard procedures^32^ revealed no evidence for violations of model assumptions. A Bland–Altman plot was used for visual evaluation of the agreement between predicted TNC-VCa CT numbers and empirical TNC-VCa CT numbers. ROC curve analysis was conducted to assess the diagnostic performance of VP-VCa CT attenuation values in detecting osteoporosis. The area under the ROC curve (AUC) and corresponding 95% confidence intervals were calculated using a non-parametric, bootstrapping-based approach^33^. The cut-off value showing the best diagnostic performance was determined and the corresponding sensitivity and specificity were reported.

## Results

### Study population

The total sample included 42 women and 55 men with a mean age of 63.2 (SD 14.1) years. Further descriptive information on the sample is summarized in Table 1. Most of the CT examinations were performed for oncological reasons (see Table 2 for further details).

**Table 1:** Patient characteristics.

	Total sample (n = 97)
Age (years)	
* M* (*SD*)	63.2 (14.1)
Height (cm)	
* M* (*SD*)	172.3 (8.6)
Body weight (kg)	
* M* (*SD*)	82.8 (22.3)
BMI (kg/m^2^)	
* M* (*SD*)	27.9 (6.9)
Sex (n (%))	
Female	42 (43.3%)
Male	55 (56.7%)
Bone mineral density (n (%))^a^	
Normal	66 (68.0%)
Osteoporosis	31 (32.0%)

^a^Bone mineral density was categorized according to Pickhardt et al.^8^ using a CT attenuation threshold of 110 HU or less in TNC to diagnose osteoporosis.

*BMI* body mass index, *M* mean, *SD* standard deviation.

**Table 2:** Clinical information on CT indications.

	n (%)
Oncological follow-up	35 (36.1%)
Suspected infection, inflammation, or abscess	16 (16.5%)
Suspected cancer disease	12 (12.4%)
Initial staging of cancer disease	12 (12.4%)
Work-up of liver cirrhosis	10 (10.3%)
Vascular indications	5 (5.2%)
Work-up for suspected acute abdominal disease in in-patients	4 (4.1%)
Other^a^	3 (3.1%)
Total	97 (100.0%)

^a^Including suspected stenosis of pancreaticojejunostomy, suspected pancreas rejection, evaluation of hepatic cyst.

### Interrater reliability

The ICCs for the two readers’ measurements ranged between 0.95 and 0.96 for all reconstructions indicating an excellent interrater reliability. A detailed listing of ICCs with corresponding confidence intervals is (Table 3)**.**

**Table 3:** Intra-class correlation coefficients for measures of bone mineral density of L1.

		ICC [95% CI]	
		True non-contrast aquisition	Venous phase post-contrast acquisition
Reconstruction	Conventional	0.96 [0.94; 0.97]	0.96 [0.93; 0.97]
	VNCa 100	0.95 [0.93; 0.96]	0.95 [0.92; 0.96]
	VNCa 25	0.96 [0.95; 0.97]	0.96 [0.94; 0.98]
	VNC	–	0.96 [0.94; 0.97]

*CI* confidence interval, *ICC* intra-class correlation coefficient, *L1* first lumbar vertebra, *VNC* virtual non-contrast images reconstructed from post-contrast venous phase acquisitions, *VNCa25* virtual non-calcium images, suppression index 25, *VNCa100* virtual non-calcium images, suppression index 100.

### Primary objectives: comparison of VCa L1 CT attenuation values from non-contrast and contrast-enhanced acquisitions

**Figure 2:**
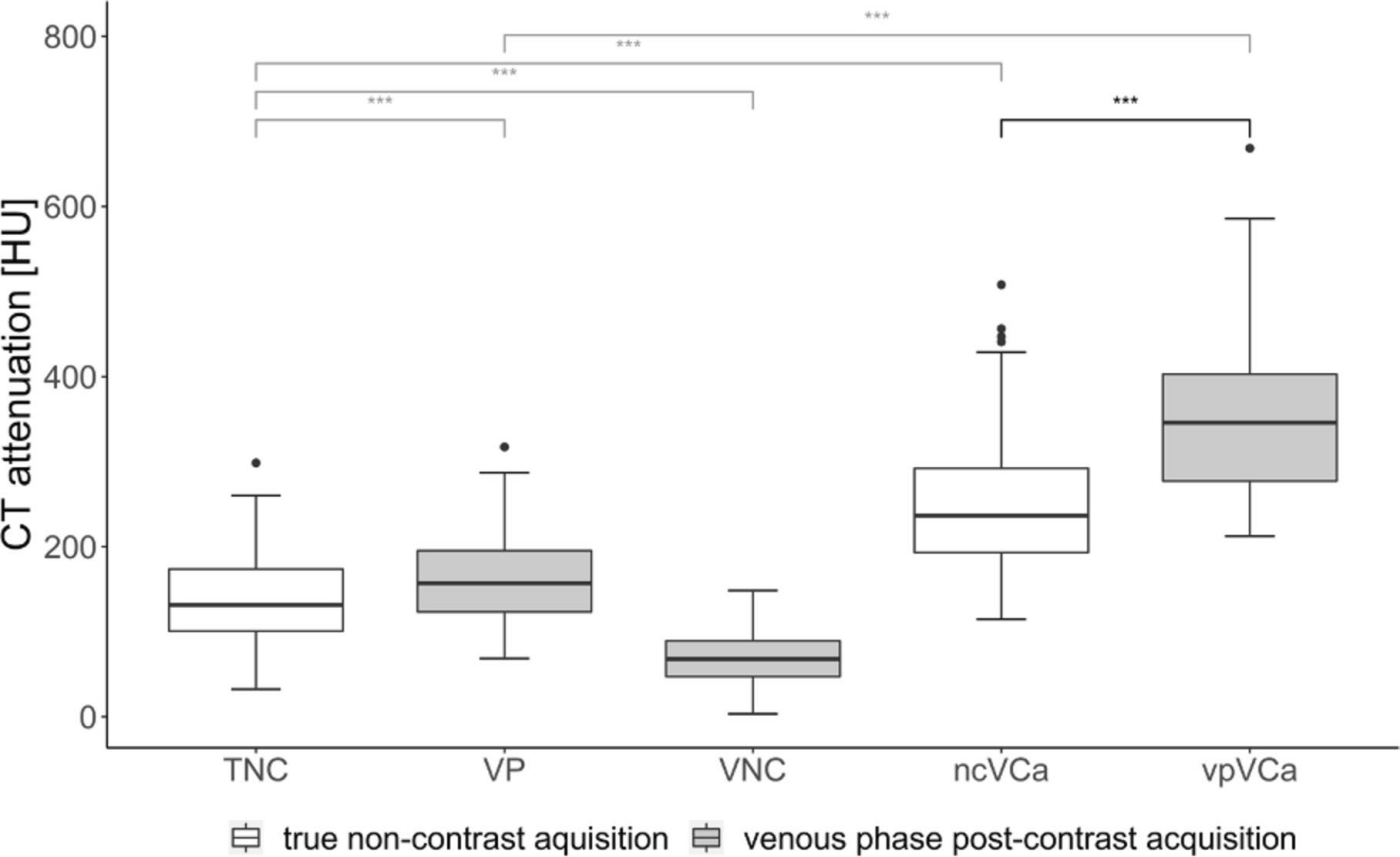
Boxplots of mean L1 CT attenuation values for different reconstructions. The brackets indicate the results of Wilcoxon Signed-Rank tests for the comparison of mean L1 CT attenuation values of TNC, VP, VNC, TNC-VCa and VP-VCa (black bracket = primary objective; grey brackets = secondary objectives). *TNC-VCa* virtual calcium-only images of true non-contrast acquisitions, *TNC* conventional images reconstructed from non-contrast acquisitions, *VNC* virtual non-contrast images reconstructed from post-contrast venous phase acquisitions, *VP* conventional images reconstructed from post-contrast venous phase acquisitions, *VP-VCa* virtual calcium-only images of post-contrast venous phase acquisitions. ***p < 0.001.

**Figure 3:**
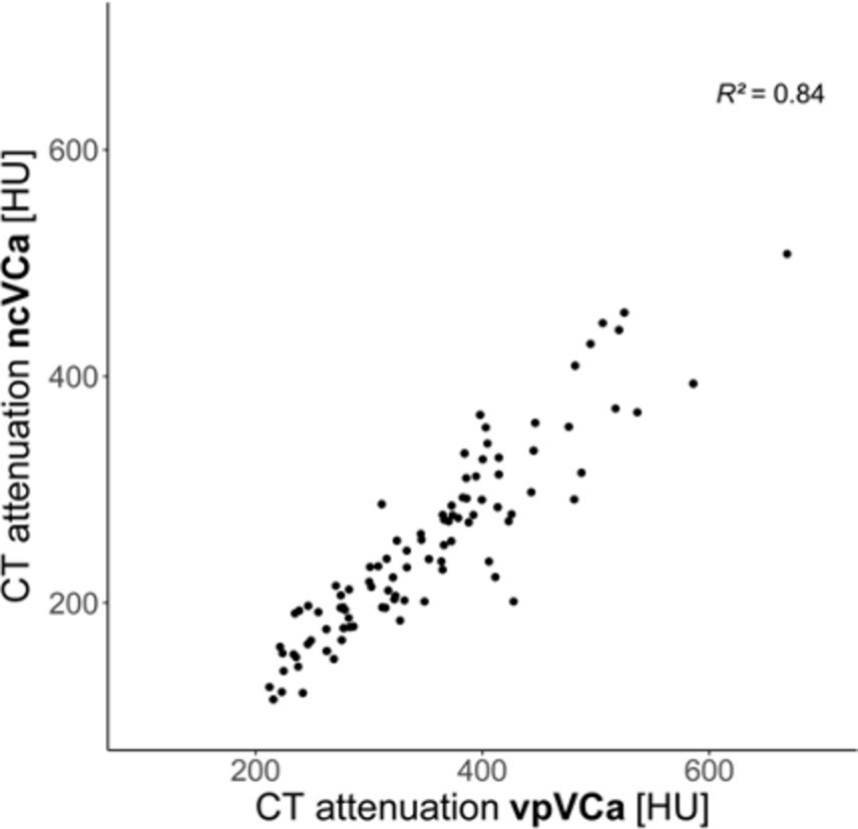
Scatterplot of CT attenuation values from VP-VCa (virtual calcium-only images of post-contrast venous phase acquisitions).

**Figure 4:**
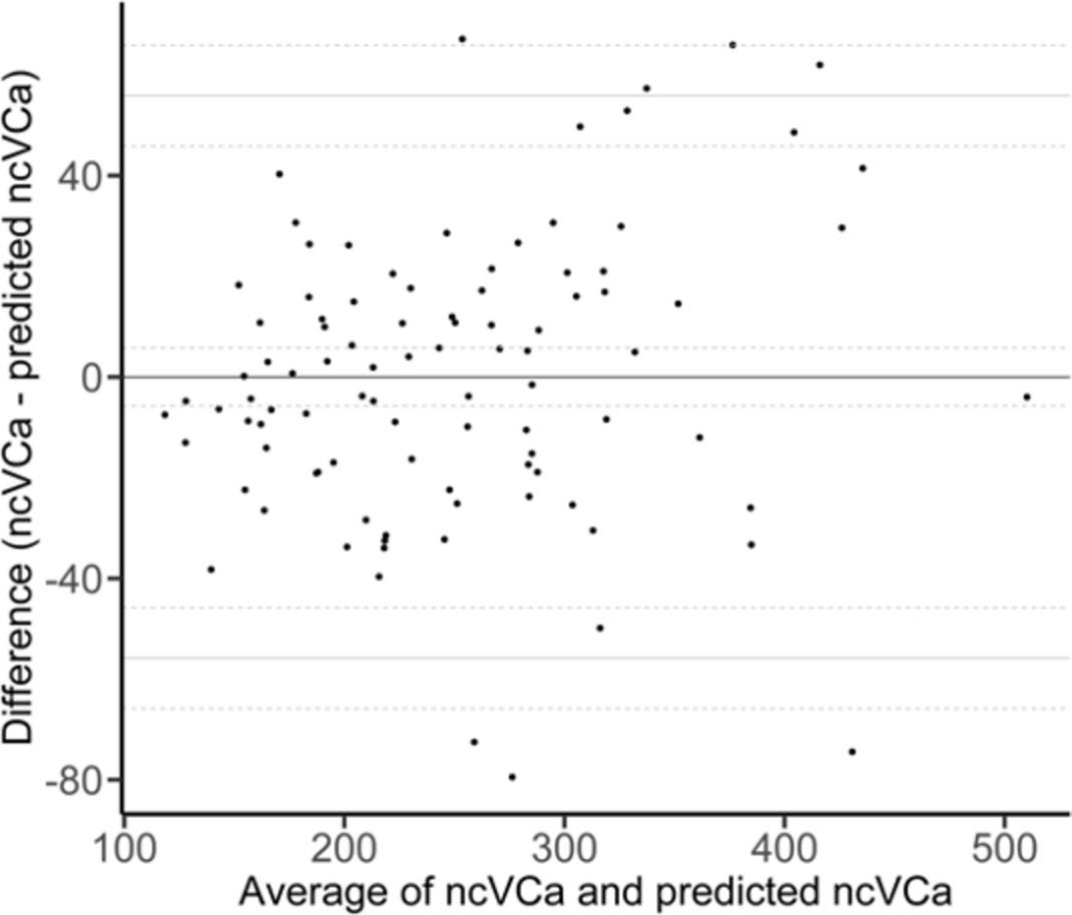
Bland–Altman plot for the comparison of observed and predicted TNC-VCa L1 CT attenuation values. *TNC-VCa* virtual calcium-only images of true non-contrast acquisitions.

Mean, median (Mdn) and corresponding measures of dispersion of L1 CT attenuation values from all reconstructions are summarized in Table 4. CT attenuation values were significantly higher in VP-VCa compared to TNC-VCa (Mdn = 345.70 HU vs. Mdn = 236.55 HU, *p* < 0.001, *r* = 0.61) (Fig. 2). Univariate regression analysis revealed a strong linear association between TNC-VCa and VP-VCa CT attenuation values (*R*^2^ = 0.84; Fig. 3). In multiple regression analysis patients’ body weight was shown to significantly improve the prediction of TNC-VCa CT attenuation values. Sex and age were eliminated from the model. The final regression equation for the prediction of TNC-VCa CT attenuation values was: _p_TNC-VCa [HU] = − 6.81 + 0.87 × VP-VCa [HU] − 0.55 × body weight [kg]. Visual assessment of the Bland–Altman plot indicated a good agreement between predicted and observed TNC-VCa CT attenuation values when applying the final regression model with most of the differences ranging within the 95% limits of agreement (− 55.85 HU to 55.85 HU) (Fig. 4).

**Table 4:** Average L1 CT attenuation values [HU].

	Reconstruction	Mean (SD)	Median (10% quantile; 90% quantile)
True non-contrast aquisition	TNC	138.4 (51.4)	131.6 (79.6; 203.4)
	VCa	250.4 (83.0)	236.6 (156.6; 361.8)
Venous phase post-contrast acquisition	VP	162.9 (50.7)	156.9 (104.5; 230.0)
	VNC	68.9 (29.0)	68.0 (35.1; 101.5)
	VCa	350.7 (91.8)	345.7 (238.2; 481.2)

*L1* first lumbar vertebra, *SD* standard deviation, *TNC* conventional images reconstructed from non-contrast acquisitions, *VCa* virtual calcium-only images, *VNC* virtual non-contrast images reconstructed from post-contrast venous phase acquisitions, *VP* conventional images reconstructed from post-contrast venous phase acquisitions.

### Secondary objectives: comparison of L1 CT attenuation values between further reconstructions

TNC CT attenuation values were significantly higher than VNC CT attenuation values (Mdn: 131.55 HU vs. 67.95 HU, *p* < 0.001, *r* = 0.61) and significantly lower than VP L1 CT attenuation values (Mdn: 131.55 HU vs. 156.9 HU, *p* < 0.001, *r* = 0.61) and TNC-VCa L1 CT attenuation values (Mdn: 131.55 HU vs. 236.55 HU, *p* < 0.001, *r* = 0.61). Accordingly, VP CT attenuation values were significantly lower than VP-VCa CT attenuation values (Mdn: 156.90 HU vs. 345.70 HU, *p* < 0.001, *r* = 0.61) (Fig. 2).

### Secondary objectives: detecting osteoporosis using L1 CT attenuation values from VP-VCa

**Figure 5:**
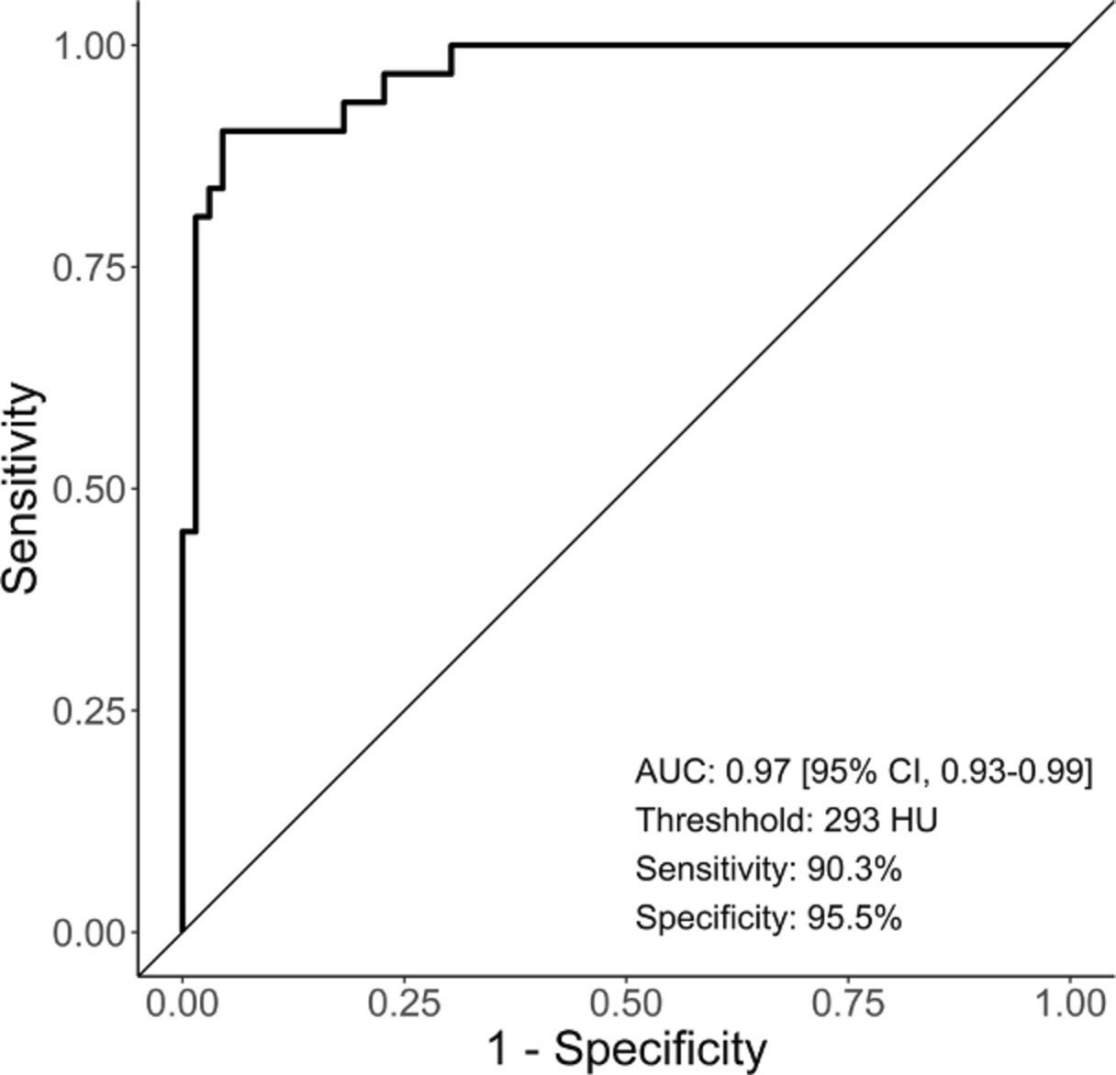
ROC curve for the detection of osteoporosis using L1 CT attenuation values obtained from VP-VCa. *AUC* area under the receiver operating characteristic curve.

ROC analysis revealed an AUC of 0.97 [95% CI 0.93–0.99] for the detection of osteoporosis using VP-VCa L1 CT attenuation values. A threshold of 293 HU was identified showing the best overall diagnostic performance and yielded a sensitivity of 90.3% and a specificity of 95.5% (Fig. 5).

## Discussion

The results of the present study showed that the injection of contrast media is accompanied by higher VCa CT attenuation values of the lumbar spine compared to CT attenuation values obtained from VCa of true non-contrast acquisitions. When not taken into account, this effect might lead to a systematic overestimation of bone density within DLCT-based osteoporosis screening with VCa. However, using the formula derived from multiple regression analysis, L1 CT attenuation values from VP-VCa could be used to predict L1 CT attenuation values from TNC-VCa. Furthermore, a L1 CT attenuation threshold of 293 HU was 90% sensitive and 96% specific for diagnosing osteoporosis in VP-VCa and could be used to identify high-risk patients with low bone mass directly, using VCa from venous phase post-contrast acquisitions.

The opportunity to reliably predict TNC-VCa CT numbers from VP-VCa CT numbers enables the application of the former proposed cut-off value by Do et al. of 126 HU in TNC-VCa to identify osteoporosis or osteopenia in the setting of contrast-enhanced CT acquisitions. However, a comparison of diagnostic performance markers between the threshold proposed by Do et al. for TNC-VCa and the threshold we found for VP-VCa in the present study, implies that VP-VCa might be as appropriate as TNC-VCa for a CT-based osteoporosis screening. According to the study by Do et al., a threshold of 126 HU was 90% sensitive and 47% specific in detecting osteoporosis in TNC-VCa. The threshold we found for VP-VCa also yielded a sensitivity of 90%, but a specificity of more than 95%. Therefore, since sensitivity was equivalently high for both thresholds, TNC-VCa and VP-VCa might perform well in clinical settings, in which patients with high risk for osteoporosis must be detected reliably and false-negative rate is supposed to be minimized. Moreover, considering the high specificity and therefore, low false-positive rate, the threshold we propose for VP-VCa L1 CT attenuation might also be well suited for patient cohorts with low risk for osteoporosis and might help avoiding unnecessary further diagnostic examinations of patients with normal BMD.

The findings of this study are in line with previous studies suggesting that the application of CM results in a systematic overestimation of CT-derived BMD^23–25^. Hence, CT attenuation values of osseous structures obtained from non-contrast and contrast-enhanced acquisitions cannot be readily compared and, most importantly, thresholds suggested for non-contrast acquisitions might not be applicable to contrast-enhanced acquisitions, and vice versa. To overcome this issue a recent study by Ding et al. investigated whether L1 CT attenuation values measured in DLCT-derived VNC are comparable to L1 CT attenuation values measured in TNC^25^. In line with the results from our study, they found L1 CT attenuation values to be significantly lower in VNC compared with TNC. The reconstruction of VNC from spectral CT data is based on material decomposition whereby the specific spectral properties of iodine are utilized to select and virtually remove the amount of attenuation which is due to iodinated material like contrast media. Since the spectral properties of iodine and bone minerals like calcium are similar, the algorithm for VNC reconstruction might not only remove iodinated material but also some of the bone minerals^25^. This line of reasoning could also explain the difference we found between L1 CT attenuation values obtained from TNC-VCa and VP-VCa. The reconstruction of VNCa, on which VCa are based on, follows the same principles as the reconstruction of VNC, but with calcium being subtracted instead of iodine. Considering the similar spectral properties of calcium and iodine, it seems reasonable that CT attenuation values of osseous structures obtained from VP-VCa are higher than those obtained from TNC-VCa since VP-VCa CT numbers might also include attenuation from iodinated contrast media. In support of these explanations, we found patients body weight to be relevant when predicting TNC-VCa CT attenuation values from VP-VCa CT attenuation values. In our study the participants received an individually tailored CM bolus which was proportional to their body weight. Since body weight is included in the regression equation as a negative term, for patients with higher body weight, i.e., patients who received a greater amount of contrast media, there is a bigger difference between VP-VCa and predicted TNC-VCa CT attenuation values. To conclude, it might be possible that the difference between TNC-VCa and VP-VCa CT attenuation values that we observed in this study was caused by a confounding of iodine- and calcium-containing structures within the reconstruction of VCa images from spectral CT data. Further studies are needed to investigate the role of different CM injection parameters on calcium-based imaging in greater detail.

Lastly, CT attenuation values in VCa of non-contrast and contrast-enhanced acquisitions was found to be higher than CT numbers from corresponding conventional reconstructions (TNC and VP). These results are in line with previous study findings that SECT-based measures of BMD are constantly lower than BMD-measures obtained from DECT and therefore support the assumption that bone mass might be underestimated in conventional SECT in a systematic manner^15,19^. Measuring CT attenuation of trabecular bone using SECT data, bone and soft tissue are confounded which increases the risk of measurement errors, especially due to changes in bone marrow composition. Therefore, DECT-based methods for BMD-quantification like virtual calcium-only imaging, allowing to control for soft tissue components, might be more suitable for a CT-based opportunistic osteoporosis screening than conventional SECT-based approaches. The present study has some limitations. Firstly, as former research results suggest that CT-derived BMD can vary systematically for different scanner types and acquisition protocols, the generalizability of our findings might be limited^34–37^. This might especially apply to different CM protocols since our data point out that the amount of CM that is injected might play a crucial role for the absolute difference between CT attenuation values of osseous structures from non-contrast and contrast-enhanced acquisitions. Furthermore, since DXA results were not available for our study cohort, we defined the presence of osteoporosis according to a CT attenuation threshold from the literature^11^ that is still controversial. Lastly, the analyses in this study were confined to the L1 vertebra, precluding comparisons with other vertebrae or combinations thereof. Further studies should incorporate various scanner types and contrast media protocols, utilize DXA results as the reference standard, and include BMD measurements of additional target regions to address these limitations.

In conclusion, this study showed that VCa can be used for an accurate assessment of bone mass in the context of contrast-enhanced DLCT acquisitions. Therefore, the results complement former research findings on virtual calcium-only imaging and support the assumption that the implementation of VCa reconstructions within CT-based osteoporosis screenings might help to improve their diagnostic accuracy. To facilitate the interpretation of BMD-measurements in VP-VCa a regression model for the reliable prediction of CT attenuation values from VCa of true non-contrast acquisitions was established. Furthermore, a separate L1 CT attenuation threshold of 293 HU for the detection of osteoporosis in VP-VCa is suggested.

## Data Availability

The datasets generated and/or analysed during the current study are not publicly available due to patient data protection but are available from the corresponding author on reasonable request.

## Abbreviations

AUC: Area under the ROC curve
BMD: Bone mineral density
CM: Contrast media
CTDI_vol_: Volumetric computed tomography dose index
DECT: Dual-energy CT
DLCT: Dual-layer spectral detector CT
DXA: Dual-energy X-ray absorptiometry
ICC: Intra-class correlation coefficient
L1: First lumbar vertebra
Mdn: Median
TNC-VCa: VCa reconstructed of true non-contrast acquisitions
ROC: Receiver operating characteristic
SBI: Spectral base images
SECT: Single-energy CT
SD: Standard deviation
TNC: True non-contrast acquisitions
VCa: Virtual calcium-only images
VNC: Virtual non-contrast image data reconstructed from post-contrast venous phase acquisitions
VNCa100: Virtual non-calcium images, suppression index 100
VNCa25: Virtual non-calcium images, suppression index 25
VP: Conventional images reconstructed from post-contrast venous phase acquisitions
VP-VCa: VCa of post-contrast venous phase acquisitions
